# Improving Thermal Support in Very and Extremely Low Birth Weight Infants during Interfacility Transport

**DOI:** 10.1097/pq9.0000000000000170

**Published:** 2019-04-15

**Authors:** Emily M. McNellis, Amy R. Leonard, Kimberly A. Thornton, Kristin C. Voos

**Affiliations:** From the * Pediatrics/Division of Neonatology, Children’s Mercy Kansas City; † the University of Missouri-Kansas City School of Medicine; ‡ Department of Transport, Children’s Mercy Kansas City, Kansas City, Mo.; § Washington Regional Medical Center, Fayetteville, Ark.; ‖ University Hospitals Cleveland Medical Center, UH Rainbow Babies and Children’s Hospital, Cleveland, Ohio

## Abstract

**Introduction:**

Review of very low birth weight (VLBW) and extremely low birth weight (ELBW) neonates transported by our specialized pediatric/neonatal transport team revealed hypothermia in up to 52% of admissions. This project aimed to decrease the incidence of hypothermia in VLBW and ELBW neonates requiring transport between facilities from 52% to <20% over 1 year.

**Methods:**

In response to gaps in knowledge and barriers to care revealed by a survey administered to transport personnel, we used a standard quality improvement plan-do-study-act model to introduce new equipment and a comprehensive thermoregulation protocol via standardized education. The primary outcome measure was the incidence of hypothermia (axillary temperature < 36.5°C) in transported VLBW and ELBW neonates. The process measure was compliance with the protocol. The balancing measures were unintended hyperthermia and transport team ground time. Transport personnel were updated on progress via meetings and run charts.

**Results:**

We reduced the incidence of hypothermia to 17% in 1 year. Compliance with the protocol improved from 60% to 76%. There was no increase in unintended hyperthermia (5% preintervention, 4% intervention, 7% surveillance, *P* = 0.76) or transport team ground time (in hours) (1.2 ± 0.9 preintervention versus 1.3 ± 0.8 intervention versus 1.2 ± 0.7 surveillance, *P* = 0.2).

**Conclusions:**

Quality improvement methods were used to develop an evidence-based, standardized approach to thermal support in VLBW and ELBW neonates undergoing transport between facilities. Following the implementation of this approach, we achieved the desired percent decrease in the incidence of hypothermia.

## INTRODUCTION

Providing a thermoneutral environment is a cornerstone of neonatal care.^1–3^ Hypothermia (central/axillary temperature < 36.5°C)^1^ is common in very low birth weight (VLBW; <1,500 g) and extremely low birth weight (ELBW; <1,000 g) neonates following birth and admission to an intensive care unit.^4–7^ Hypothermia in this population can complicate care by impairing glucose utilization/metabolism, immune function, and coagulation. More importantly, hypothermia has consistently been associated with increased mortality.^4–6^ As hypothermia is a potentially modifiable risk factor for significant morbidity and mortality, several guidelines, and successful quality improvement (QI) efforts toward improving thermoregulation in VLBW/ELBW neonates have been developed.^1–3,8–13^ Most studies and QI projects, however, have concentrated on the “inborn” neonate within the delivery room, newborn nursery, and neonatal intensive care unit. Unlike these settings, the transport environment is further challenged by limited monitoring, time constraints, rapidly changing ambient temperature, and other unseen variables. At this time, transport-specific guidelines for maintaining a thermoneutral environment are lacking.^14^

As part of a comprehensive thermoregulation QI initiative in our intensive care nursery, review of admission temperatures in VLBW and ELBW neonates transported by our specialized pediatric/neonatal transport team revealed hypothermia in up to 52% of admissions. A root cause analysis identified gaps in staff education, transport operations, and application of current evidence-based thermoregulation practice. This QI project aimed to decrease the incidence of hypothermia in VLBW and ELBW neonates requiring interfacility transport from 52% to <20% over 1 year without increasing the incidence of unintended hyperthermia and transport team ground time with the patient.

## METHODS

This project was approved by the Children’s Mercy Hospital (CMH) Institutional Review Board as QI.

### Setting and Patient Population

CMH is a freestanding quaternary care children’s hospital operating a 78-bed Level IV neonatal intensive care unit, the CMH Intensive Care Nursery (CMH-ICN). The CMH-ICN admits an average of 800–900 patients per year, of whom 75%–80% are outborn. The Children’s Mercy Critical Care Transport (CMCCT) team performs an average of 5,000 interfacility transports per year, with about 20% of those being for neonatal patients. Modes of transport include ambulance, fixed wing, and rotor wing servicing a region with about a 200-mile radius. In addition to these modes of transport, about one-fifth of the VLBW/ELBW transports performed by the team are done by foot between a neighboring delivery hospital, Truman Medical Center, across an enclosed “link” connecting Truman Medical Center and CMH. The team is composed of a registered nurse (RN), respiratory therapist (RT), and emergency medical technician (EMT).

The patient population included neonates <1,500 g transported by the CMCCT team and admitted to the CMH-ICN between July 2012 and June 2015. We excluded VLBW/ELBW neonates transported by any other transport team from analysis. The study covers 3 periods including preintervention (July 2012–June 2013), intervention (July 2013–June 2014), and a surveillance period (July 2014–June 2015).

### Preintervention

A task force including team members from both the CMH-ICN and CMCCT was formed and met at least bimonthly during the preintervention and intervention periods. The task force completed a root cause analysis by distributing an anonymous electronic survey (via Survey Monkey) to the RN, RT, and EMT transport personnel of CMCCT. This survey was constructed with the aim of assessing general thermoregulation knowledge, familiarity with existing transport protocols, and barriers to provide thermal support during transport. A thorough evaluation of our existing protocols and a literature review were also conducted to compare our practices with published guidelines and recommendations for thermoregulation in the VLBW/ELBW population.

The response rate to the electronic survey was 44%. Although the survey results revealed adequate knowledge of basic thermoregulation concepts, we identified several barriers to thermal support. These barriers included: (1) lack of continuous temperature monitoring capability; (2) hypothermia of neonates occurring before transport team arrival to the referring hospital; and (3) thermal support as a lower priority due to other pressing clinical conditions and time constraints. We also identified gaps in our protocols about the most current guidelines. These gaps included: (1) inconsistent temperature range targets across several protocols; (2) lack of guidance for prewarming the transport incubator and other equipment anticipated to come in contact with the neonate; and (3) lack of guidance for interventions should hypothermia develop. Three other barriers were identified as potentially modifiable causes of hypothermia: (1) hypothermia developing during umbilical line placement (due to placement of sterile towels that impede radiant heat in radiant warmers); (2) the influence of cabin temperature during transport; and (3) delivery of nonheated, nonhumidified air in neonates receiving noninvasive and invasive mechanical respiratory support.

### Measurements

The primary outcome measure was the incidence of hypothermia (temperature < 36.5°C) upon admission in transported VLBW/ELBW neonates. We assessed this measurement by an axillary temperature taken by the transport team at the time of patient handoff before transfer of the patient from the transport incubator to a radiant warmer or incubator in the CMH-ICN. The process measure was transport team compliance with the implemented thermoregulation protocol. The balancing measures were unintended hyperthermia (axillary temperature > 37.5°C) and transport team ground time (interval between team arrival at referral bedside and departure with the patient).

### Intervention

**Fig. 1.**
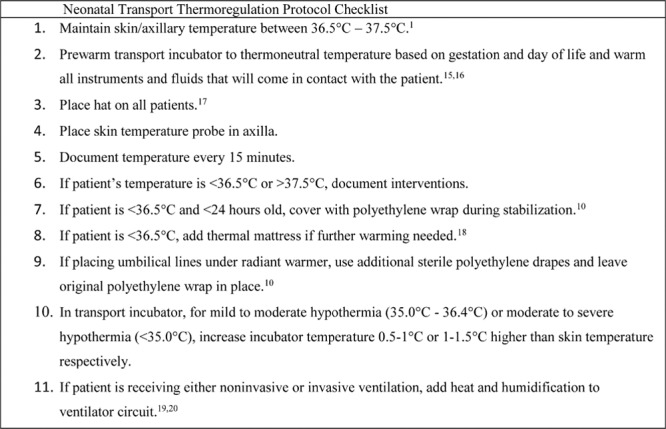
Guidelines and interventions included in the Neonatal Transport Thermoregulation Protocol with primary evidence-based references.

After evaluating the barriers and educational and operational gaps, we performed the following interventions. In April 2013, continuous temperature monitoring was made possible by the introduction of the Propaq MD (Zoll Medical Corporation, San Jose, Calif.) with the STS-400 Level 1 Skin Temperature Sensor (Smiths Medical, St. Paul, Minn.). Use of this new equipment obviated the need to open the incubator portholes, which disrupts temperature regulation periodically. The probe was placed in the axilla and temperatures were recorded every 15 minutes and at the time of admission. Between February and May 2013, we developed a dedicated neonatal transport thermoregulation protocol aligning guidelines and interventions with existing best evidence (Fig. 1^15-18^). Before implementation of this protocol in July 2013, all RN, RT, and EMT personnel received standardized education sessions following their mandatory preshift safety briefs between May and June 2013. These education sessions included: (1) sharing the preintervention incidence of hypothermia in VLBW/ELBW transferred by the team, thereby raising awareness of the local problem; (2) describing the aim of the QI project; (3) providing a basic thermoregulation didactic; (4) providing an in-service for new equipment; (5) outlining the thermoregulation protocol in detail; and (6) discussing expectations for documentation of axillary temperature, ambient temperature (both incubator and cabin), and interventions if the patient was outside of the desired temperature range. During this time, we also provided education to referral hospitals via regional nurse manager meetings and CMCCT Transport educational outreach visits. In August of 2013, the NeoPod-T (Westmed, Inc., Tucson, Ariz.) was also introduced to deliver heated and humidified air to neonates that require high flow nasal cannula, continuous positive airway pressure, or invasive mechanical ventilation. As part of the thermoregulation protocol, we also introduced the practice of utilizing a sterile polyethylene drape as the sterile field under a radiant warmer for neonates requiring umbilical line placement in the standardized education sessions.

### Surveillance

During the surveillance period, we shared results of the intervention and provided a “brush up” education session to the teams during two quarterly meetings. A checklist was also devised summarizing the thermoregulation protocol and attached to the transport incubators. Temperatures continued to be collected prospectively and reviewed every month.

### Data Collection, Statistical Analysis, and Data Sharing

Data collected included demographics, temperature, and transport variables and were analyzed using Excel (Microsoft Office 2007, Redmond, Wash.) and SAS software (SAS Institute Inc., Cary, N.C.). Continuous variables and discrete data were analyzed using Student *t* test and chi-squared test, respectively. Analysis of variance was used to determine if there were differences in baseline demographic, temperature, and transport variables among the 3 periods. Run charts were generated in Excel and used to track percent neonates with hypothermia (<36.5°C axillary) upon admission, aggregate percent compliance with the transport thermoregulation protocol, and percent of neonates with hyperthermia. These charts were shared with the team quarterly up to March 2014, then monthly starting April 2014 through June 2015. Control charts were generated with QI Macros (KnowWare International Inc., Version 2015.10, Denver, Colo.).

## RESULTS

### Demographic, Temperature, and Transport Comparisons

A total of 84, 79, and 77 neonates met inclusion criteria during the preintervention, intervention, and surveillance periods, respectively (Table 1). Gestational age and weight did not differ between the groups, but the preintervention group had a higher percentage of patients transported at <24 hours of age (76% preintervention versus 58% intervention versus 53% surveillance, *P* = 0.006). The preintervention group had a lower mean admission temperature (36.1 ± 1.3°C preintervention versus 36.8 ± 0.6°C intervention versus 36.9 ± 0.5°C surveillance, *P* = <0.0001). Mean rendezvous time (arrival of the transport team to the referral bedside following initial transport request) in hours did not differ among the groups (2.1 ± 3.5 preintervention versus 1.9 ± 2.6 intervention versus 2 ± 1.8 surveillance, *P* = 0.3), but mean transport time (time between leaving the referral facility to arrival at the CMH-ICN) was shorter in the preintervention group (0.5 ± 0.5 preintervention versus 0.9 ± 0.7 intervention versus 0.8 ± 0.7 surveillance, *P* = 0.003). There was a difference in the distribution of transport modes, with the preintervention group having a lower fixed wing and higher rotor wing and “link” transports compared to the other 2 groups (*P* = 0.03).

**Table 1.**
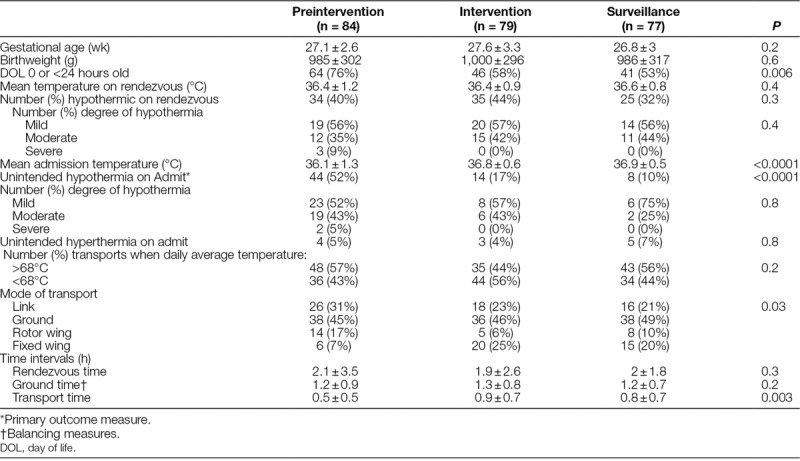
Clinical, Demographic, Temperature, and Transport Data

### Primary Outcome, Process, and Balancing Measures

**Fig. 2.**
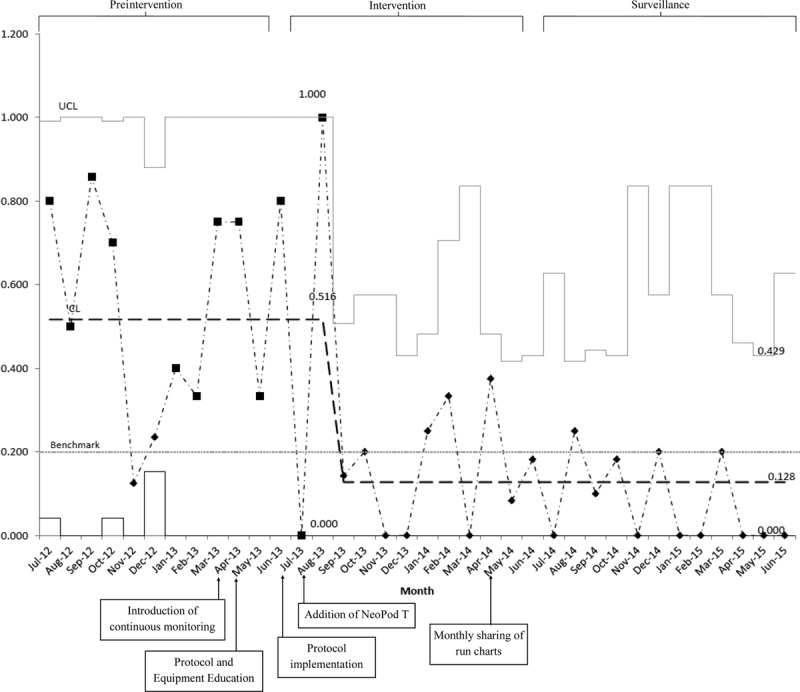
Control chart displaying proportion of transported ELBW/VLBW neonates admitted with hypothermia during consecutive months of the study (July 2012–June 2013: Preintervention, July 2013–June 2014: Intervention, July 2014–June 2015: Surveillance). Arrows signify the timing of interventions. The mean for hypothermia (dashed line), the 20% benchmark (dotted line), and upper and lower 3 σ control limits are included.

Hypothermia on admission in transported VLBW/ELBW neonates (primary outcome) was reduced from 52% during preintervention to 17% following the intervention and further fell to 10% during the surveillance period (*P* ≤ 0.0001) (Fig. 2). Admission hypothermia has remained *<*10% since this time (July 2015–June 2016 [10%] and July 2016–June 2017 [4%]). At this time, no further changes to the protocol have been made. The percent protocol compliance (process measure) rose from 60% to 76% during the intervention and surveillance periods, respectively. Unintended hyperthermia (balancing measure) on admission remained unchanged (5% pre, 4% intervention, 7% surveillance, *P* = 0.76) and transport ground time in average hours (balancing measure) did not differ among the groups (1.2 ± 0.9 preintervention versus 1.3 ± 0.8 intervention versus 1.2 ± 0.7 surveillance, *P* = 0.2).

### Development of Hypothermia during Transport

The mean temperature of patients at rendezvous did not differ among the groups (36.4 ± 1.2°C preintervention versus 36.4 ± 0.9°C intervention versus 36.6 ± 0.8°C surveillance, *P* = 0.4) nor did the percentage of patients that were hypothermic (40% pre, 44% intervention, and 32% surveillance, *P* = 0.3). Although there was not a significant difference in the distribution of mild, moderate, and severe hypothermia at rendezvous (*P* = 0.4) nor at admission (*P* = 0.8) among the groups, only the preintervention group had 2 patients who experienced severe hypothermia (temperature < 32°C).

Following the protocol implementation, fewer patients who were initially normothermic at rendezvous became hypothermic during transport (55% preintervention versus 29% intervention versus 25% surveillance), and more patients who were initially hypothermic became normothermic by admission (18% preintervention versus 42% intervention versus 50% surveillance). Outcomes were similar even when analyzing only patients with transport times <1 hour in each group. There was an improvement in avoiding hypothermia when patients were initially normothermic (30% preintervention versus 8% intervention versus 6% surveillance) and achieving normothermia by admission when hypothermic at rendezvous (13% preintervention versus 27% intervention versus 19% surveillance).

### Potential Risk Factors for Hypothermia during Transport

Because the preintervention period differed from the other periods with shorter mean transport time and more patients <24 hours old, we analyzed key demographic- and transport-specific variables as potential risk factors and for their influence on hypothermia in each group. Evaluation of patients with hypothermia at admission among all time periods (n = 66) revealed hypothermia was more likely if transport time was <1 hour (*P* = 0.0003), the patient was <24 hours old (*P* = 0.005), hypothermia was present on rendezvous (*P* = 0.007), and transport occurred during the warmer months of the year (average daily high temperature > 68°C)^21^ (*P* = 0.02). Variables that were not associated with increased admission hypothermia included rendezvous time >1 hour (*P* = 0.7), ground time >1 hour (*P* = 0.7), level of referral facility (*P* = 0.3), or mode of transport (*P* = 0.2). For those variables associated with admission hypothermia, no specific variable appeared to affect the rate of hypothermia disproportionately during any period (Table 2). Furthermore, improvements remained significant excluding <1 hour transport times (preintervention admission hypothermia 31% versus intervention 11% versus surveillance 3%, *P* = 0.03) and patients <24 hours old (preintervention admission hypothermia 45% versus intervention 12% versus surveillance 3%, *P* = 0.0001).

**Table 2.**
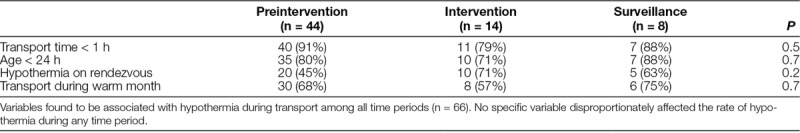
Risk Factors for Hypothermia during Transport

## DISCUSSION

Maintaining a continuum of effective therapy from the delivery room to Neonatal Intensive Care Unit (NICU) admission presents additional barriers in the event of an “outborn” neonate requiring interfacility transport to a higher level of care.^2,22–25^ Hypothermia is a frequent complication and is associated with increased morbidity and mortality in the VLBW/ELBW neonate.^4–7^ In response to a high incidence of hypothermia in our transported VLBW/ELBW neonates, we utilized standard QI methods to develop an evidence-based standardized approach to thermal support during transport.

Birth centers aiming to decrease admission hypothermia in VLBW/ELBW neonates following delivery have reported achieving an incidence as low as 0%–10% after implementing standardized, evidence-based practices.^11,12^ Unlike the delivery room setting where efforts can be taken to avoid hypothermia, in the transport setting, the team frequently assumes care of hypothermic patients on rendezvous (32%–44% incidence in this study) and faces rapidly changing ambient temperatures among other variables that challenge maintaining a thermoneutral environment. Applying current evidence-based practices from stationary settings and determining appropriate thresholds for improvement, therefore, had to be made with these variables in mind.

Guidelines for the rate of rewarming the hypothermic VLBW/ELBW are lacking.^26^ Gradual rewarming by 0.5C°/h was used throughout all periods as it is currently recommended for patients with both intended and unintended hypothermia to avoid complications such as shock, seizures, and arrhythmias.^27–29^ Thus, we chose a threshold of <20% incidence of admission hypothermia for the primary outcome measure to account for a potentially irreducible number of patients that would not achieve normothermia (if originally hypothermic) due to short transport times. The threshold proved to be a conservative estimate, however, as maintenance of normothermia and achieving normothermia in initially hypothermic patients both improved throughout the study regardless of the length of transport.

Initial stabilization of pediatric and neonatal patients before interfacility transport has been suggested to improve outcomes as opposed to a “scoop and run” approach.^2,23,30^ Yet, a point of diminishing return exists with longer ground times, delaying definitive care.^23^ With this concern in mind, we chose ground time as a balancing measure due to the potential of this interval being prolonged once the thermoregulation protocol was introduced. Our study showed no difference in average ground time among periods. However, this result suggests that the additional interventions can be successfully streamlined into the care of clinically complex patients without delaying transport. Unintended hyperthermia was also chosen as a balancing measure as it, too, has associated morbidities^1,31^ and should be avoided. There was no increased incidence of hyperthermia throughout the periods.

Documentation, although improved, is still inconsistent and is a problem that additional QI initiatives are addressing. Regardless, the most salient albeit intangible improvement observed was increased team awareness that effective thermal support is critical to the global care of the neonate.^8^ Before our interventions, both temperature monitoring and thermal interventions were low priorities. Presenting thermal support as a longitudinal effort and hypothermia as a source of clinical instability most likely set the foundation for the improvements observed.

One unexpected observation of the study was the improvement seen during transports <1 hour. Short transports were found to be a risk factor for hypothermia regardless of rendezvous temperature. Following protocol implementation, however, fewer patients that were initially normothermic became hypothermic and more patients that were initially hypothermic became normothermic by the admission in this subgroup. This improvement challenged the assumption that hypothermia in the setting of a short transport is due only to insufficient time to rewarm a hypothermic patient or that there would be insufficient time for the normothermic patient to become hypothermic. Instead, the study suggests transport practice is an important variable in maintaining thermoregulation in VLBW/ELBW patients as opposed to time and initial temperature at rendezvous alone. Another intriguing observation was the effect the season had on the team’s ability to maintain a thermoneutral environment. Paradoxically, the risk of admission hypothermia was increased during warmer as opposed to cooler months of the year underscoring the greater influence cabin temperature has on thermoregulation as opposed to seasonal temperature. These observations have led to timely warming/rewarming interventions at rendezvous, increased vigilance even during the briefest of geographical transitions and tighter control of the ambient ambulance, rotor wing, and fixed-wing cabin temperatures. Similarly, these are variables that continue to be targeted in delivery rooms and stationary NICU settings^3,9,11,12,32^ and have so far been applied to all modes of transport.

Although we have observed significant improvements by applying thermoregulatory practices from “inborn” settings, some approaches may not be appropriate for transport. For example, the effectiveness of a polyethylene wrap left in place under a radiant warmer or incubator following delivery and short transfer to the admitting NICU has been well documented.^10^ Rewarming or maintaining temperature, however, in an incubator with a convective mode of warming during a prolonged interfacility transport may be hindered if the wrap is left in place. Although some patients in our study remained wrapped following initial stabilization, this was not evaluated as a risk factor for admission hypothermia, but should be in future analyses to guide practice. Rewarming rates have also been informed by observation of hypothermia in adults and pediatric patients and more recently by the research protocols of therapeutic hypothermia in neonates with encephalopathy.^27–29^ A faster rewarming rate (>0.5°C/h) has been suggested to have no increased risk in poor neonatal outcomes and may actually decrease the incidence of respiratory distress syndrome in the ELBW population.^26^ Such a change may ameliorate the increased risk for morbidity and mortality that VLBW/ELBW neonates requiring interfacility transport have compared with their inborn cohorts.

Available equipment designed for the transport environment also continues to be a limiting factor in optimizing thermoregulation. Providing heat and humidification of inspired gas during noninvasive and invasive mechanical ventilation is one of these challenges. This aspect of care should receive pointed attention as heat and humidification of inspired gas decrease the incidence of hypothermia and other respiratory morbidities.^19,20^ Heat and humidification of inspired gas has been recommended in the delivery room^33^ and is used as an ancillary treatment for core-warming in the treatment of established hypothermia.^34^ Independent study of its effectiveness and management within the transport workflow will need to be carried out.

Educational outreach is also an important effort toward improving thermoregulation in VLBW/ELBW neonates requiring interfacility transport. Although not statistically significant, a decline in the incidence of hypothermia occurred at the referral facilities throughout this QI project. We have therefore continued to emphasize this aspect of pretransport care through regional S.T.A.B.L.E.^2^ courses provided by transport personnel, neonatal nurse practitioners, and neonatologists.

## CONCLUSIONS

Standard QI methods were utilized to develop an evidence-based standardized approach to thermal support during interfacility transport of VLBW/ELBW neonates. Although the evidence-based practices were gleaned from “inborn settings,” we achieved and sustained a decrease in admission hypothermia to <10%. Further development of comprehensive guidelines specific to the transport setting is needed.

## ACKNOWLEDGMENTS

The authors wish to thank the following individuals for their assistance with this manuscript: Ashley K. Sherman, MA, for statistical analysis; Amber L. Bellinghausen for editorial assistance; and James D. McBrien for editorial assistance.

## DISCLOSURE

The authors have no financial interest to declare in relation to the content of this article.

## References

[1] World Health Organization (WHO), Safe Motherhood. Thermal Protection of the Newborn: A Practical Guide. 1997 Geneva, Switzerland: World Health Organization;

[2] Karlsen KA The S.T.A.B.L.E. Program Pre-Transport Post-Resuscitation Stabilization Care of Sick Infants: Guidelines for Neonatal Healthcare Providers. 2013 6th ed Salt Lake City, Utah: S.T.A.B.L.E., Inc;

[3] American Heart Association, American Academy of Pediatrics. Neonatal Resuscitation Textbook. 2016 7th ed Washington, D.C.: American Academy of Pediatrics and American Heart Association;

[4] Costeloe K Hennessy E Gibson AT The EPICure study: outcomes to discharge from hospital for infants born at the threshold of viability. Pediatrics. 2000;106:659–671. 11015506 10.1542/peds.106.4.659

[5] Laptook AR Salhab W Bhaskar B; Neonatal Research Network. Admission temperature of low birth weight infants: predictors and associated morbidities. Pediatrics. 2007;119:e643–e649. 17296783 10.1542/peds.2006-0943

[6] Miller SS Lee HC Gould JB Hypothermia in very low birth weight infants: distribution, risk factors and outcomes. J Perinatol. 2011;31(suppl 1):S49–S56. 21448204 10.1038/jp.2010.177

[7] Chang HY Sung YH Wang SM Short- and long-term outcomes in very low birth weight infants with admission hypothermia. PLoS One. 2015;10:e0131976. 26193370 10.1371/journal.pone.0131976 PMC4507863

[8] Lyon A Applied physiology: temperature control in the newborn infant. Current Paediatrics. 2006;16:386–392.

[9] Knobel R Holditch-Davis D Thermoregulation and heat loss prevention after birth and during neonatal intensive-care unit stabilization of extremely low-birthweight infants. Adv Neonatal Care. 2010;10(suppl 5):S7–S14. 20838082 10.1097/ANC.0b013e3181ef7de2

[10] McCall EM Alderdice F Halliday HL Interventions to prevent hypothermia at birth in preterm and/or low birthweight infants. Cochrane Database Sys Rev. 2008:CD004210 doi: 10.1002/14651858.CD004210 10.1002/14651858.CD004210.pub3 18254039

[11] Manani M Jegatheesan P DeSandre G Elimination of admission hypothermia in preterm very low-birth-weight infants by standardization of delivery room management. Perm J. 2013;17:8–13. 24355884 10.7812/TPP/12-130 PMC3783084

[12] Pinheiro JM Furdon SA Boynton S Decreasing hypothermia during delivery room stabilization of preterm neonates. Pediatrics. 2014;133:e218–e226. 24344110 10.1542/peds.2013-1293

[13] Lapcharoensap W Lee HC Temperature management in the delivery room and during neonatal resuscitation. Neoreviews. 2016;17:e454–e462.

[14] Leng H Wang H Lin B Reducing transitional hypothermia in outborn very low birth weight infants. Neonatology. 2016;109:31–36. 26485388 10.1159/000438743

[15] Scopes JW Ahmed I Minimal rates of oxygen consumption in sick and premature newborn infants. Arch Dis Child. 1966;41:407–416. 21032442 10.1136/adc.41.218.407 PMC2019504

[16] Scopes JW Ahmed I Range of critical temperatures in sick and premature newborn babies. Arch Dis Child. 1966;41:417–419. 21032443 10.1136/adc.41.218.417 PMC2019509

[17] Laptook AR Watkinson M Temperature management in the delivery room. Semin Fetal Neonatal Med. 2008;13:383–391. 18501693 10.1016/j.siny.2008.04.003

[18] Chawla S Amaram A Gopal SP Safety and efficacy of trans-warmer mattress for preterm neonates: results of a randomized controlled trial. J Perinatol. 2011;31:780–784. 21527905 10.1038/jp.2011.33

[19] te Pas AB Lopriore E Dito I Humidified and heated air during stabilization at birth improves temperature in preterm infants. Pediatrics. 2010;125:e1427–e1432. 20457686 10.1542/peds.2009-2656

[20] Restrepo RD Walsh BK; AARC Clinical Practice Guideline. Humidification during invasive and noninvasive mechanical ventilation: 2012. Respir Care. 2012;57:782–788. 22546299 10.4187/respcare.01766

[21] Intellicast. The Authority in Expert Weather. Available at: http://www.intellicast.com/Local/History.aspx?. Accessed online July 1, 2018.

[22] Mori R Fujimura M Shiraishi J Duration of inter-facility neonatal transport and neonatal mortality: systematic review and cohort study. Pediatr Int. 2007;49:452–458. 17587267 10.1111/j.1442-200X.2007.02393.x

[23] Section on Transport Medicine, American Academy of Pediatrics. Guidelines for Air and ground Transport of Neonatal and Pediatric Patients. 2007 3rd ed Elk Grove Village, Il: American Academy of Pediatrics;

[24] Lasswell SM Barfield WD Rochat RW Perinatal regionalization for very low-birth-weight and very preterm infants: a meta-analysis. JAMA. 2010;304:992–1000. 20810377 10.1001/jama.2010.1226

[25] Arora P Bajaj M Natarajan G Impact of interhospital transport on the physiologic status of very low-birth-weight infants. Am J Perinatol. 2014;31:237–244. 23690051 10.1055/s-0033-1345259

[26] Rech Morassutti F Cavallin F Zaramella P Association of rewarming rate on neonatal outcomes in extremely low birth weight infants with hypothermia. J Pediatr. 2015;167:557–561.e1. 26168772 10.1016/j.jpeds.2015.06.008

[27] Azzopardi D Brocklehurst P Edwards D; TOBY Study Group. The TOBY Study. Whole body hypothermia for the treatment of perinatal asphyxial encephalopathy: a randomised controlled trial. BMC Pediatr. 2008;8:17. 18447921 10.1186/1471-2431-8-17 PMC2409316

[28] Scaravilli V Bonacina D Citerio G Rewarming: facts and myths from the systemic perspective. Crit Care. 2012;16(suppl 2):A25.

[29] Schmutzhard E Fischer M Dietmann A Rewarming: facts and myths from the neurological perspectives. Critical Care. 2012;16(S2):A24.

[30] Gardner SL Carter BS Enzman-Hines MI S.T.A.B.L.E Pretransport Stabilization of the Newborn. Merenstein and Gardner, Eds: Merenstein and Gardner’s Handbook of Neonatal Intensive Care. 2016:8th ed St. Louis, Mo.: Elsevier; 39–40.

[31] Impey LW Greenwood CE Black RS The relationship between intrapartum maternal fever and neonatal acidosis as risk factors for neonatal encephalopathy. Am J Obstet Gynecol. 2008;198:49.e1–49.e6. 18166304 10.1016/j.ajog.2007.06.011

[32] American Academy of Pediatrics. In: American College of Obstetric Practice, ed. Guidelines for Perinatal Care. 2012 7th ed Washington, D.C.: American Academy of Pediatrics;

[33] Wykoff MH Aziz K Escobedo MV Part 13: neonatal resuscitation 2015 American Heart Association guidelines update for cardiopulmonary resuscitation and emergency cardiovascular care. Pediatrics; 2015 136(S2):S196–S218. 26471383 10.1542/peds.2015-3373G

[34] Mekjavić IB Eiken O Inhalation rewarming from hypothermia: an evaluation in -20 degrees C simulated field conditions. Aviat Space Environ Med. 1995;66:424–429. 7619035

